# Pazopanib-associated remodeling of platelet-immune cell crosstalk and immune suppressive platelet-derived extracellular vesicles in metastatic RCC

**DOI:** 10.3389/fimmu.2025.1696460

**Published:** 2026-01-07

**Authors:** Gianpiero Lupoli, Stefano Bergamini, Jeannette Salsetta, Alessandro Mereu, Agata Cova, Elisa D’Angelo, Eriomina Shahaj, Elisabetta Vergani, Martina Stroscia, Emma Di Carlo, Licia Rivoltini, Elena Verzoni, Veronica Huber

**Affiliations:** 1 Translational Immunology Unit, Fondazione IRCCS Istituto Nazionale dei Tumori, Milan, Italy; 2 Department of Medicine and Sciences of Aging, PhD Program in “Molecular Oncology and Tumor Immunology”, “G. d’Annunzio University” of Chieti-Pescara, Chieti, Italy; 3 Genetics and Genomic Medicine Research & Teaching Dept, University College London GOS (UCL GOS) Institute of Child Health, London, United Kingdom; 4 Medical Oncology Department, Fondazione IRCCS Istituto Nazionale dei Tumori, Milan, Italy

**Keywords:** antiangiogenics, TKI, RCC, immune suppression, extracellular vesicles, platelets

## Abstract

**Background:**

Antiangiogenics promote immune activation by reducing myeloid-derived suppressor cells (MDSCs) and enhancing natural killer (NK) and T cell functions in metastatic RCC patients. However, these effects are transient, leading to compensatory immunosuppression. Platelets (PLT) and their extracellular vesicles (PLT-EVs) modulate immune and angiogenic pathways, suggesting a role in immune reprogramming during therapy.

**Methods:**

Circulating EVs were longitudinally profiled in metastatic RCC patients (n=8) undergoing Pazopanib therapy. EVs, isolated by differential ultracentrifugation from baseline, 3- and 6-month plasma samples, were characterized by bead-based multiplex assay and nanoparticle tracking analysis. Results were correlated with blood counts, RNA-seq and flow cytometry immune profiles.

**Results:**

Pazopanib induced temporally structured EV compartment alterations. After three months, EVs were enriched in immune markers (CD8, CD56, CD19, CD1c, HLA-DR), consistent with immune activation, whereas PLT-derived markers (CD41b, CD42a, CD29) were diminished. By six months, PLT-EV markers recovered, with CD62P^+^ and CD29^+^ EVs co-expressing immunoregulatory and angiogenic molecules (CD209, CD105). PLT-EV abundance correlated with the expansion of regulatory T cells (Tregs), PD-L1^+^ monocytes and MDSCs, together with suppression of NK cells. PLT activation and PDGF signaling pathways decreased in PBMC from patients with clinical benefit.

**Conclusions:**

Despite the small sample size and absence of functional experiments, our results suggest that Pazopanib promotes cytotoxic immune programs but, by six months, reprograms PLT-EVs towards different adhesion characteristics contributing to Treg and MDSC expansion while suppressing NK activity. PLT-EVs may influence the balance between immune activation and suppression during anti-angiogenic therapy, suggesting PLT-EVs as biomarkers and therapeutic targets in mRCC.

## 1 Introduction

Renal cell carcinoma (RCC), while accounting for only 2-3% of adult cancers, poses a significant mortality burden in its metastatic form (mRCC) (1, 2). Blockade of VEGFR-mediated angiogenic signaling with tyrosine kinase inhibitors (TKIs), such as Pazopanib, has become standard of care in the treatment of mRCC (3–5). As TKI monotherapy and TKI ± immunotherapy (IO) combinations now represent the predominant first-line therapeutic strategies in mRCC, increasing attention has been directed toward identifying biomarkers predictive of treatment response. In this context Xu et al. identified FHL2 as a potential predictor of immune checkpoint inhibitor (ICI) plus TKI therapy (6). In addition to their vascular effects, VEGFR-TKIs-induced stress can push inflammatory cell-death programs (PANoptosis) (7), leading to immune remodeling and immunosuppression relief. In fact, VEGFR-TKIs promote dendritic cell maturation, constrain myeloid-derived suppressor cells (MDSCs), enhance NK cell cytotoxicity and effector T cell function (8–10). However, these immune-activating effects are often transient. Clinical and preclinical studies suggest that an initial wave of cytotoxic immune activation may later give rise to compensatory immunosuppressive programs, raising the hypothesis that treatment-induced changes in circulating mediators may actively contribute to reprogramming immune responses over time (9–11). Among the mediators, extracellular vesicles (EVs) represent active players in reprogramming immune and metabolic pathways, such as TGF-β/lncRNA axis, as evidenced in macrophage and myocarditis models, where natural or engineered EV cargo delivery determines macrophage metabolism and immune tone (12–14).

Platelets (PLT) and platelet-derived extracellular vesicles (PLT-EVs) can interact with both tumor and immune cells. Elevated PLT counts and high PLT-to-lymphocyte ratio (PLR) are associated with poor prognosis in RCC and other cancers (15, 16), representing potential non-invasive biomarkers. Similarly, C-reactive protein-to-albumin ratio and perinephric fat correlate with prognosis and inflammation in non-metastatic clear cell RCC (ccRCC) (17).

PLT can directly promote the proliferation, invasion and metastasis of cancer cells, as well as angiogenesis, chemoresistance and immune evasion (18, 19). PLT-EVs, especially large extracellular vesicles (LEVs), are released predominantly upon activation and represent near the 50% of plasma EVs (20), with documented pro-angiogenic and pro-metastatic behavior (21, 22). Similarly to other extracellular vesicles (EVs) also PLT-EVs function as intercellular messengers, deliver proteins, lipids and RNAs that can modulate angiogenesis, immune responses and therapy resistance (23). In RCC, tumor-derived and stromal EVs can facilitate immune evasion by suppressing NK cell functions, including receptor expression, degranulation and cytokine production (24). PLT-EVs carry adhesion molecules such as integrins (e.g., αIIbβ3) and P-selectin (CD62P) (25, 26), immune checkpoint molecules (27), growth factors (e.g., TGF-β) (28), checkpoint ligands such as PD-L1 (29, 30) an other immune and vascular signaling components (31). Adhesion molecules, which represent the most abundant proteins in EVs (32), determine their targeting: integrin β1 (CD29) promotes EV uptake into recipient immune cells (33, 34), while P-selectin mediates binding to PSGL-1 on leukocytes (35–37). EVs bearing CD105, DC-SIGN (CD209) or CD40 can further bias immune programs toward angiogenesis, tolerance or altered antigen presentation, depending on cellular context (38–42). PLT GARP/TGF-β complexes induce Tregs and suppress NK cytotoxicity (43, 44). Thus, PLT-EVs could represent potential mediators of Pazopanib-induced immune reprogramming. However, how VEGFR-TKI therapy dynamically reshapes the composition and function of circulating PLT-EVs and whether these changes contribute to the shift from early immune activation to later immune suppression, remains to be elucidated. By integrating longitudinal plasma EV phenotypes with PLT counts, PBMC flow cytometry and transcriptome profiles, we investigated the contribution of PLT-EVs to immune regulation under anti-angiogenic therapy.

## 2 Materials and methods

### 2.1 Patient and healthy donors

Patients were recruited at the Fondazione IRCCS Istituto Nazionale dei Tumori, Milan, Italy. All had metastatic clear cell renal cell carcinoma and received first-line treatment with Pazopanib according to clinical practice. Pazopanib was administered orally at a standard dose of 800 mg once daily for at least six months (9, 45). Patients (n=8; responders n=6 and non-responders n=2 classified according to RECIST criteria (45) and healthy donors (n=3) provided written informed consent under a protocol approved by the INT Ethical Committee [INT146/14, INT48/21].

### 2.2 Blood collection

Plasma was obtained from whole peripheral blood collected at baseline and at the third and sixth months of therapy. Peripheral blood was processed within one hour of collection. Blood samples were centrifuged at room temperature (RT) for 10 min at 2.000 x g. The collected plasma was then centrifuged a second time at RT for 10 min at 2.000 x g prior to storage at -80°C.

### 2.3 Isolation of the EVs

EVs were isolated from plasma by differential centrifugation, following the established MISEV guidelines (46). Thawed plasma samples were subjected to centrifugation at 2.000 × g for 15 min at RT. After discarding the pellet, the samples were centrifuged at 10.000 × g for 45 min at RT to pellet large EVs (LEVs). The resulting supernatant was then transferred into 1.5 mL ultracentrifuge tubes (Thermo Scientific) and ultracentrifuged at 100.000 × g for 2 h at 4°C, using a TLA100.3 in an ultracentrifuge Optima Max XP (both Beckman Coulter). The pellet containing small EVs (SEVs) was collected and resuspended in PBS filtered at 0.1 µm, followed by a second ultracentrifugation step at 100.000 × g for 2 h at 4°C. After the washing step, the supernatant was discarded and the SEV-containing pellet collected and resuspended in PBS filtered at 0.1 µm. To keep freeze–thaw cycles at a minimum (47), Bradford protein determination and NTA were performed before storage of EV samples at -80°C for further analysis.

### 2.4 Determination of the size and concentration of the EVs

The size and concentration of EVs were determined by nanoparticle tracking analysis (NTA) using a NanoSight NS300 instrument (Alfatest) according to manufacturer’s instructions. Three 60-second videos were recorded for each sample at camera level 16, with a detection threshold of 5. The syringe pump was set to a flow rate of 30 µl/s. Samples were diluted in filtered PBS (0.1 µm) to obtain a concentration between 20 and 120 particles per frame.

### 2.5 Bead-based flow cytometry analysis of EVs

After thawing, EV samples were subjected to EV surface marker profiling using a MACSPlex EV Kit IO, human (Miltenyi Biotec) according to manufacturer’s instructions. Briefly, EVs (10 µg of the total protein, as determined by the Bradford assay) were incubated with antibody-coated MACSPlex EV Capture Beads, microspheres coated with antibodies specific for 37 membrane epitopes and 2 isotype controls (CD45, CD3, CD4, CD8, CD56, CD19, HLA-DR/DP/DQ, CD2, CD105, CD1c, CD25, CD49e, ROR1, CD209, SSEA-4, HLA-A/B/C, CD9, CD81, CD63, CD40, CD62P, CD11c, MCSP, CD146, CD41b, CD42a, CD24, CD86, CD44, CD326, CD133-1, CD29, CD69, CD142, CD31, CD20, CD14, RAE control, mIgG1 control). Bound EVs were then labeled with MACSPlex EV detection reagents containing CD9, CD63, and CD81 antibodies, forming a sandwich complex between capture bead, EV and detection reagent. Positive signals indicated the presence of specific EV surface markers. Data acquisition was performed on a CytoFLEX S flow cytometer (Beckman Coulter). Median fluorescence intensity (MFI) values were used to quantify surface marker expression, and data were analyzed with Kaluza software (Beckman Coulter). Results of the control sample (capture beads only) were subtracted from the raw data for each marker, causing the appearance of negative/zero values for different markers in the LEV fraction, which were marked as “not detected” as recommended by the manufacturer’s instructions. Then, normalization on EV-specific tetraspanins was obtained by dividing the resulting values by the mean of CD9, CD81, CD63 values.

### 2.6 Gene set enrichment analysis

Normalized data matrices have been taken from the NCBI Gene Expression Omnibus database (http://www.ncbi.nlm.nih.gov/geo/) under accession number GSE146163 (9). From a total of 47,323 probes arrayed on the Illumina HT12v4 beadchip, probes targeting multiple genes were collapsed by averaging their expression intensities, resulting in a final dataset comprising 12,913 unique genes. All analyses were performed using R (version 4.4.1; RStudio Inc.). Differential expression analyses between groups (Responders vs. Non-Responders) were conducted at baseline, three months, and six months post-treatment using unpaired t-tests. Functional enrichment analyses were performed using the clusterProfiler R package (48). Gene Ontology (Biological Process) enrichment was evaluated, and pathways with FDR adjusted p-value below 0.05 were considered significantly enriched. Among the enriched terms, pathways associated with major immune cell populations and immune response processes were specifically isolated for further interpretation. Visualization of the enriched pathways was carried out using the ridgeplot function implemented in the enrichplot (49) (R package version 1.28.4, https://bioconductor.org/packages/enrichplot) R package.

### 2.7 Statistical analysis

Statistical analyses were performed using GraphPad Prism 10.0 (GraphPad Software Inc.) and R 4.4.1 (R Core Team (2024). *R: A Language and Environment for Statistical Computing*. R Foundation for Statistical Computing, Vienna, Austria. https://www.R-project.org/). The Friedman test was applied to MACSPlex data, two-way ANOVA to NTA data, and the Wilcoxon signed-rank test to group comparisons and all other paired datasets. For correlation analyses, Spearman’s rank correlation test was performed using the SciPy package (Python). MACSplex correlations were considered significant for an adjusted p value ≤ 0.05 (Benjamini-Hochberg), while for the other correlations a non-adjusted p value ≤ 0.05 was considered significant. Linear curve fitting and coefficient of determination (R²) values were calculated with NumPy and scikit-learn, respectively. Correlation matrices, bubble plots and scatter plots were generated using seaborn packages, while correlation network diagrams using NetworkX package (Python). Only correlation results showing a p value < 0.05 and an absolute Spearman coefficient > 0.3 were considered.

## 3 Results and discussion

### 3.1 Pazopanib alters the levels of PLT-EVs and pro-angiogenic factors in plasma of mRCC patients

**Figure 1:**
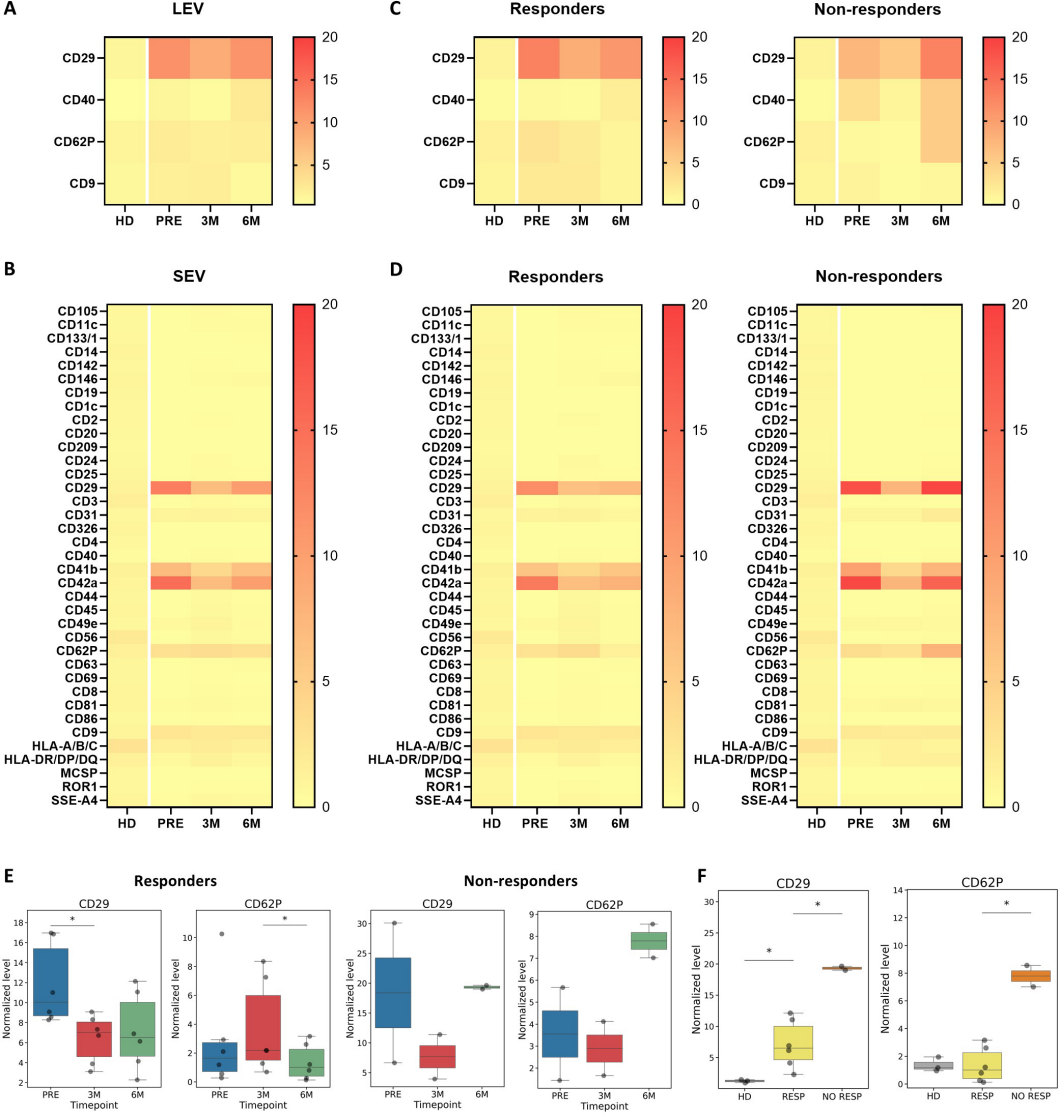
Effects of Pazopanib Treatment on EV surface markers. **(A, B)** Heatmap showing the levels of 37 EV markers on LEVs and SEVs of mRCC patients (n=8) before (PRE) and after three (3M) and six (6M) months of Pazopanib treatment, compared to healthy donors (HD, n=3). Normalized levels are shown as a gradient from yellow to red. **(C, D)** Levels of EV markers on SEVs (C) and LEVs **(D)** of responders (n = 6) and non-responders (n = 2) before and after three and six months of Pazopanib treatment, compared to healthy donors (HD, n=3). **(E)** Results of the only significant longitudinal differences of PLT markers in SEV from responders (left) and the corresponding results in non-responders (right). * p < 0.05. **(F)** Significant comparisons between responders (RESP) and non-responders (NO RESP) SEVs markers at six months. * p < 0.1.

In previous work, we demonstrated that Pazopanib treatment of mRCC patients induces changes in circulating cytotoxic lymphocyte populations through an indirect mechanism involving MDSC (9). To explore whether EVs participate in this process, we investigated the profile of EV-associated markers in the plasma of the previously analyzed patients receiving Pazopanib treatment (9). Plasma samples were obtained before therapy initiation, after three months and after six months of treatment. NTA evaluation of LEVs and SEVs, separated by sequential ultracentrifugation at 10.000 × g and 100.000 × g, respectively, revealed no relevant differences in concentration but a significantly higher SEV mode at six months of therapy (**Supplementary Figures S1A**, **S2B**). In contrast, comparison with EVs from plasma of healthy donors (HD, n=3), isolated using the same method of sequential centrifugations, showed that that SEV concentration in plasma of patients was significantly lower after three and six months Pazopanib therapy, but not at baseline, with respect to HD (**Supplementary Figure S1A**). Regarding the EV size, comparison with HD SEVs revealed both, significantly higher mean and mode in patients’ vesicles at all 3 timepoints (**Figure 1B**). These results suggest major differences in the systemic EV compartment of mRCC patients and a potential impact of the TKI on SEV release (**Supplementary Figure S1**). Surface marker expression was next assessed in LEV and SEV fractions using a MACSplex bead-based assay with 37 immune-related antigens and the canonical CD9, CD81, CD63 tetraspanin antibody cocktail. Normalization to the EV tetraspanins CD9, CD81, and CD63 revealed a consistent enrichment of CD29 in both EV fractions, together with the PLT-associated antigens CD31, CD41b, CD42a and CD62P. However, after normalization, many markers showed zero values in the LEV fraction, causing their exclusion. In SEVs, additional markers such as CD9, HLA-A/B/C, and HLA-DR/DP/DQ were also prominently detected. Longitudinal analysis of normalized EV-marker profiles revealed that CD29 expression decreased at three months and rose again by six months in both LEV and SEV fractions, while CD41b and CD42a followed the same biphasic pattern but restricted to SEVs (**Figures 1A, B**). Notably, CD29 has been found in PLT-EVs (34). Levels of these markers were also higher in patients compared to HD, independently of the timepoint (**Supplementary Figure S2**). The stratified analysis based on clinical response to the treatment revealed that in contrast to non-responders, LEVs and SEVs of responders (n=6) displayed no rebound of CD29, CD41b and CD42a (**Figures 1C–E**). Moreover, the PLT activation marker CD62P in SEVs showed an opposite trend between the two groups with a significant decrease at six months in responders compared to non-responders (n=2), suggesting an association between PLT activation and the resistance onset. CD62P levels were also comparable between responders and HD (**Figure 1F**). As VEGFR-TKI treatment is known to inhibit PLTs activation and degranulation (50, 51), the different amount of PLT-EVs between responders and non-responders may reflect an intrinsic resistance of PLTs themselves to VEGFR-TKIs, which could potentially influence the response.

**Figure 2:**
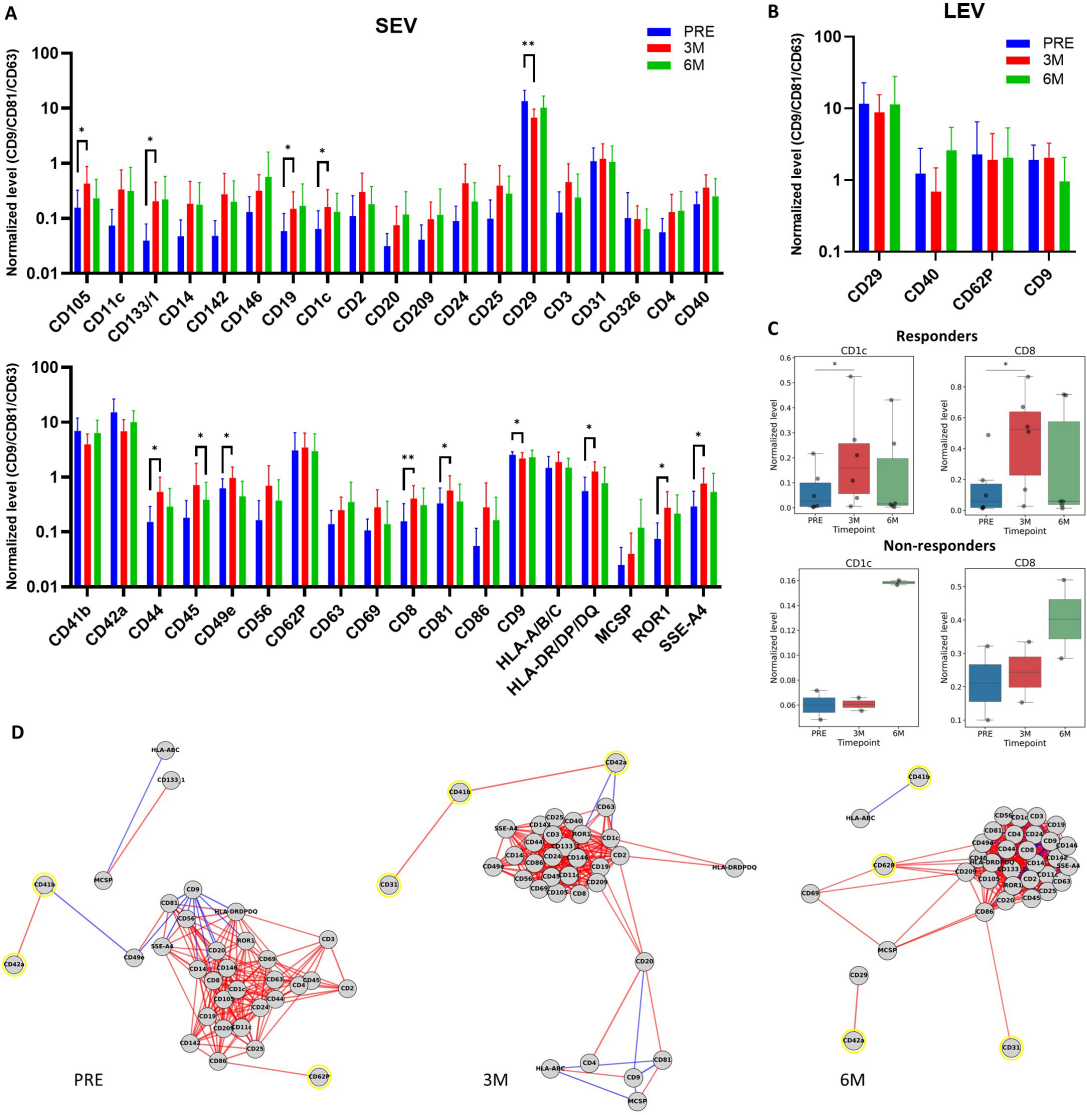
Longitudinal analysis of EV markers distribution after Pazopanib treatment. **(A, B)** Detailed analysis of changes in EV markers on SEVs **(A)** and LEVs **(B)** from all patients at three time points (PRE, 3M, 6M). The results are expressed as log_10_ of values normalized to the EV-related markers CD9, CD81, and CD63. * p < 0.05, ** p < 0.01. **(C)** Results of the only significant longitudinal differences in SEV markers in responders (upper) and the corresponding results in non-responders (lower). * p < 0.05. **(D)** Correlation network diagram showing significant correlations between SEV markers. Negative and positive correlations between nodes are shown as red and blue lines, respectively. PLT-related markers are indicated by yellow circles. Only results with q value < 0.1 are shown.

Apart from PLT-related markers, a set of immune- and progenitor-associated EV markers displayed significant fluctuations during Pazopanib treatment (**Figures 2A, B**). At the three-month timepoint, SEVs were enriched for CD105 (endoglin), CD133 (stem/progenitor cell marker), CD19 and CD1c (B cell and dendritic cell antigens), as well as CD44, CD56 and CD8 markers of cytotoxic lymphocytes and NK cell subsets. Interestingly, the significant increase of CD1c and CD8 was present only in SEVs from responders (**Figure 2C**). However, SEVs from both responders and non-responders showed a downregulation of the immunoactivation-related markers compared to HD, reflecting the overall reduced anticancer activity (**Supplementary Figure S3**). Increased levels of CD81, HLA-DR/DP/DQ, ROR1, and SSEA-4 within all patients were also observed. Among EV markers, only CD9 tetraspanin was significantly reduced at three months, while the remaining markers showed an increase at 3-month time point. By six months, most of these markers trended back toward baseline, with the exception of CD49e, an integrin subunit, which showed a significant reduction after six months therapy compared to the three-month time point, but not between baseline and three months (**Figures 2A, B**).

Pazopanib appears to induce temporally structured remodeling of the circulating EV compartment. The transient rise of immune and progenitor-related markers at three months suggests cellular activation, while the biphasic dynamics of PLT markers and CD29 point to a distinctive modulation of PLT-EVs during therapy. Following the EV remodeling observed at three months, the absent rebound of PLT-associated markers and CD29 at six months only in responders, together with the decrease of CD62P, suggests that activated PLTs and their EVs may have a role in the immunosuppressive behavior observed under Pazopanib treatment.

### 3.2 PLT-related markers correlate with other immunosuppressive markers on EVs after Pazopanib treatment

Since markers related to PLTs emerged as the most prevalent antigens in SEVs and displayed a decrease at three months followed by an increase at six months, we hypothesized that they may be co-expressed on the same vesicle populations. To address this, we performed a correlation analysis across all MACSplex markers in SEVs and LEVs. This analysis revealed a progressive increase in the complexity of marker associations over time in SEVs, with the highest number of correlations observed after six months of treatment (**Supplementary Figure S4A**), whereas LEVs showed only a single significant association between CD62P and CD29 at this later timepoint (**Supplementary Figure S4B**).

Within SEVs, PLT markers maintained positive correlations with each other prior to treatment and at three months, such as CD41b with CD42a and CD31 with CD41b, consistent with their co-release on PLT-EVs. Interestingly, by six months these intraplatelet correlations were lost, suggesting that prolonged exposure to Pazopanib reshapes the PLT-EV compartment into a more heterogeneous pool where PLT markers no longer track together (**Figure 2D**). Instead, PLT markers began to correlate with immune-regulatory and angiogenic markers. Specifically, CD62P showed a strong positive association with CD105, CD209, and CD40 at six months, while CD42a correlated positively with CD29 at this same timepoint and CD41b negatively correlated with HLA-A/B/C.

Furthermore, negative correlations between CD42a and the immune cell-derived markers CD1c and ROR1 emerged at three-months (**Supplementary Figure S5**). All the observed correlations were also confirmed in the absence of any normalization, even if there was an overall increase in associations between markers (**Supplementary Figures S6**, **S7**).

The biological implications of these associations could be relevant: CD29, which correlated positively with PLT markers in both SEVs and LEVs at six months, has been implicated in EV uptake and functional RNA delivery to recipient cells (33), is associated with poor prognosis and drug resistance in some cancer types (52, 53), and its enrichment on PLT-EVs at this timepoint may potentiate the transfer of suppressive signals to immune targets. CD105, previously shown to promote angiogenesis when carried by RCC-derived EVs (38), and CD209, a receptor expressed by M2-polarized macrophages and tolerogenic dendritic cells (39, 40), both correlated with CD62P on SEVs at six months. This points to the possibility that EVs from activated PLT may exert both pro-angiogenic and immunosuppressive properties. The additional co-expression of CD40, a molecule capable of shaping T cell responses through co-stimulatory or tolerogenic pathways depending on context (41, 42), highlights the complexity of PLT-EVs in immunomodulation.

Since CD62P is a marker of PLT activation and a mediator of interactions between PLTs, monocytes, and cancer cells (35, 54–56), its convergence with integrin β1, CD105, and CD209 suggests a scenario in which PLT-EVs at six months may act as vectors of both angiogenic and suppressive signaling.

Platelet counts follow changes in PBMC features during Pazopanib treatment.

**Figure 3:**
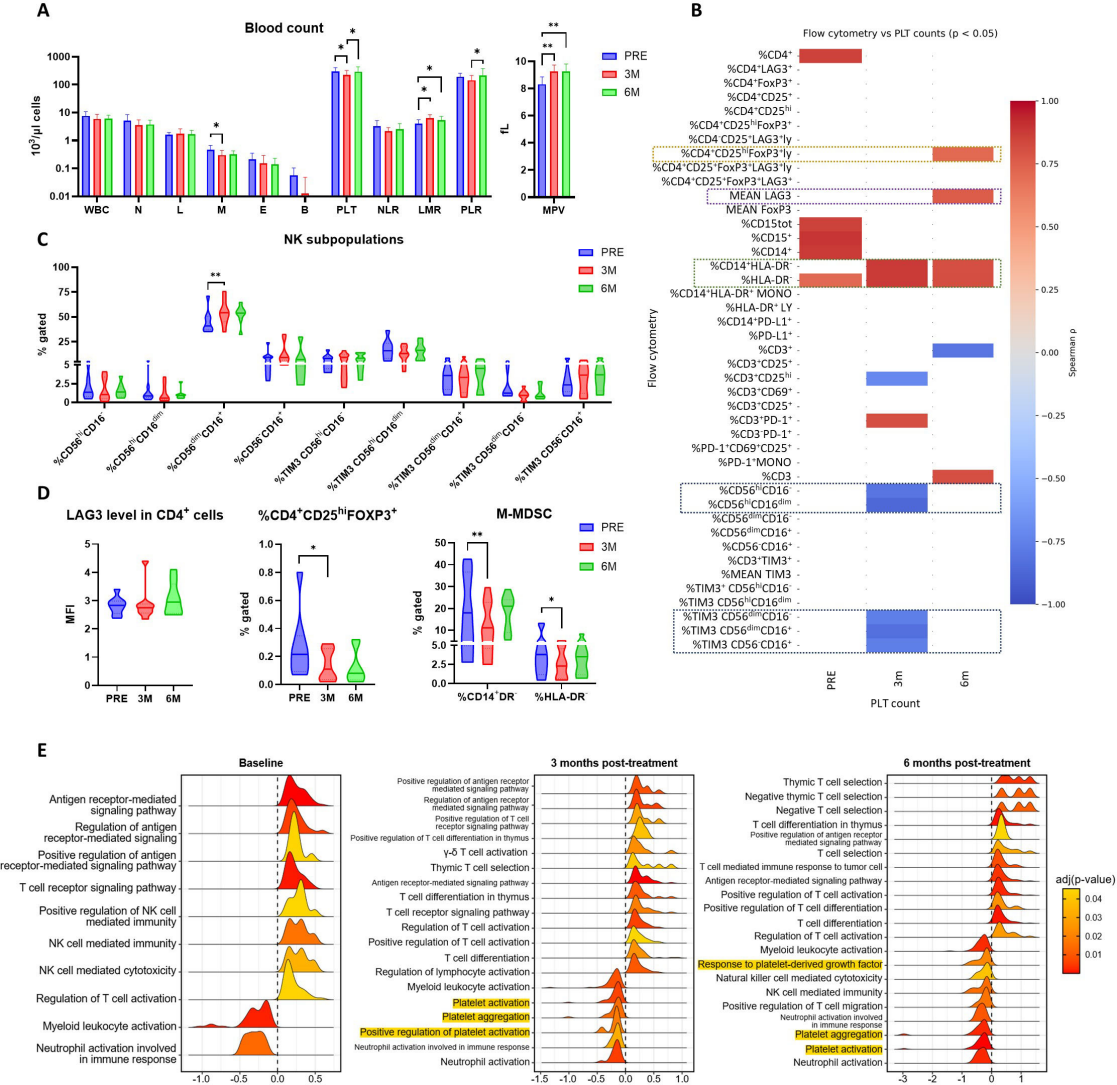
Association between PLT counts and immune cell population levels under Pazopanib treatment. **(A)** Blood cell concentrations (left) and mean platelet volume (right) from patients before (PRE) and after (3M, 6M) treatment. WBC = white blood cells, N = neutrophils, L = lymphocytes, M = monocytes, E = eosinophils, B = red blood cells, PLT = platelets, NLR = neutrophil/lymphocyte ratio, LMR = lymphocyte/monocyte ratio, PLR = platelet/lymphocyte ratio, MPV = mean platelet volume. **(B)** Matrix showing the significant Spearman correlations between platelet counts and flow cytometry. Negative and positive correlations are shown as a gradient from blue to red. Colored boxes indicate changes in correlation over time for NK populations (blue), monocytic myeloid suppressive cells (green), LAG3 MFI (violet), and regulatory T cells (yellow). **(C, D)** Flow cytometry results of the population correlated with PLT at different timepoints of treatment. NK subpopulations in CD3- lymphocytes, %CD14+CD25hiFOXP3+ in CD3+CD4+ cells, %CD14+DR- and %DR- in CD14+ cells. MFI = median fluorescence intensity. The median of the values is shown. * p < 0.05, ** p < 0.01. **(E)** Immune- and PLT-related enriched pathways in responders compared to non-responders at baseline (left), and after three (center) and six months (right) of Pazopanib treatment. Color gradient from red to yellow represents the adjusted p value. The Normalized Enrichment Score (NES) is reported as a shift in the curve from the center to the left or right. Results are sorted by NES. Platelet-related pathways are evidenced in yellow. Only results with adj p < 0.05 are visualized.

Because elevated PLT levels are associated with poor prognosis in RCC (15), we next asked whether the changes in PLT-EV markers observed in plasma were reflected by alterations in circulating PLT counts and whether these changes influenced the systemic immune cell landscape. As expected, PLT concentrations declined significantly at three months compared with the pre-treatment baseline but returned to near-baseline values after six months of Pazopanib therapy. Analogous results have been reported after treatment with sunitinib (57) and PD-1 immune checkpoint blockade-induced thrombocytopenia (58). A similar trend was observed for the PLR, with a significant increase between the third and sixth month of treatment. Conversely, the lymphocyte-to-monocyte ratio (LMR) increased at both three and six months, together with a reduction in the absolute monocyte count specifically at three months. The mean PLT volume (MPV) increased significantly after treatment and maintained the same level at six months (**Figure 3A**). An increase in MPV in mRCC patients after Pazopanib treatment has been already documented (57). However, we found no significant difference in blood counts and MPV between responders and non-responders (**Supplementary Figure S8**).

Correlation analysis revealed that PLT numbers were positively associated with total white blood cell (WBC) and monocyte counts at the three-month timepoint, but these associations were lost by six months (**Supplementary Figure S9**).

To integrate PLT dynamics with immune cell phenotypes, we re-analyzed flow cytometry data from PBMCs collected at the same timepoints (9). As previously reported, the frequency of CD14^+^ monocytes declined after three and six months of treatment, with monocytic MDSCs (CD14^+^HLA-DR^-^) specifically reduced at the three-month timepoint. In contrast, cytotoxic NK cell subsets expanded at three months. Correlating these immune data with PLT counts revealed that circulating PLT levels were positively associated with HLA-DR^-^ populations across pre-treatment, three and six months and with CD14^+^ cells at baseline and at three months (**Figure 3B**). The PLT-mediated shift of monocytes and macrophages toward immunosuppressive phenotype has been described (59). This points to a sustained positive interaction between PLT and the monocytic MDSC compartment.

In parallel, PLT counts were negatively correlated with several NK cell subsets at three months, including CD56^high^CD16^dim/-^ cells, TIM3^+^CD56^dim/-^CD16^+^ cells, and TIM3^+^CD56^dim^CD16^-^ cells, in line with the already described immunosuppressive behavior of PLTs on NK cells (60, 61). Thus, PLT reduction is associated with enhanced activity of NK effector populations at the three months timepoint when also cytotoxic activity was increased (9) (**Figure 3C**). By six months, however, PLT counts correlated positively with the frequency of CD4^+^CD25^high^FOXP3^+^ regulatory T cells and with increased expression of LAG3 within the CD4 compartment (**Figure 3D**). These findings were reproduced when considering the PLR rather than absolute PLT numbers. Interestingly, the PLT-induced increase of FoxP3^+^ T cells at the expense of the other T cell populations has been reported (62).

These results indicate that PLT dynamics under treatment not only reflect the biphasic remodeling of PLT-EVs but may also be associated with functional modulations in PBMC subsets. The transient reduction in PLT numbers at three months coincides in fact with diminished monocytic MDSCs and enhanced NK cell activity, whereas the recovery of PLT counts at six months associates with expansion of Tregs and expression of exhaustion markers. However, even if it is likely that the number of PLT takes part in the immunosuppressive behavior observed under Pazopanib treatment, this is not exclusive of non-responders.

### 3.3 Activated platelet interaction with circulating immune cells influences clinical response to Pazopanib treatment

Even if we found no association between PLT counts and therapeutic response, the levels of PLT-related markers, including the activation marker CD62P, on plasma EVs significantly diverged in the two groups after six months of treatment. We thus hypothesized that there may be a difference in the PLT activation status between groups.

Significant results of Gene Set Enrichment Analysis (GSEA) performed on 12,913 genes in PBMC from responder patients compared to non-responders were filtered for PLT- and immune-related pathways (9) (**Figure 3E**, **Supplementary Table S1**). Before treatment, we found no difference in pathways associated with PLTs between groups. However, PLT aggregation and activation pathways resulted significantly downregulated in responders at three and six months of Pazopanib treatment, while the response to PLT derived growth factor (PDGF) resulted downregulated only at six months. Interestingly, PDGF is known to foster resistance to TKI in RCC, favoring a pro-angiogenic tumor microenvironment (63). As the known inhibitory effect of Pazopanib on PLT aggregation and activation (50, 51) was significantly enhanced in responders compared to non-responders, PLT themselves could be implied in resistance onset. Notably, the resistance to EGFR-TKI induced by PLT activation has been demonstrated in lung adenocarcinoma (64).

Several immune-related pathways also differed between groups even before treatment. Specifically, antigen receptor signaling and NK cell cytotoxicity pathways were upregulated, whereas myeloid and neutrophil activation pathways were downregulated in responders compared with non-responders, highlighting intrinsic immune differences independently of therapy. Other pathways emerged only after treatment. At three and six months, T cell selection, differentiation, activation and antigen-mediated signaling were upregulated in responders, while neutrophil activation resulted downregulated. Unexpectedly, pathways related to NK cell immunity/cytotoxicity and T cell migration showed a negative score at six months, highlighting the need for further investigation.

These data demonstrate that differences in Pazopanib response are accompanied by distinct PLT activation pathways detectable within PBMCs, suggesting that activated PLT interact with circulating immune cells and influence therapeutic outcome. The downregulation of PLT activation, aggregation and PDGF signaling pathways in PBMCs from responders compared with non-responders further suggests that PLT activation may directly contribute to the establishment of immunosuppression during Pazopanib treatment.

### 3.4 The PMBC shift towards immunosuppression after treatment is followed by changes in the PLT-EVs phenotype

**Figure 4:**
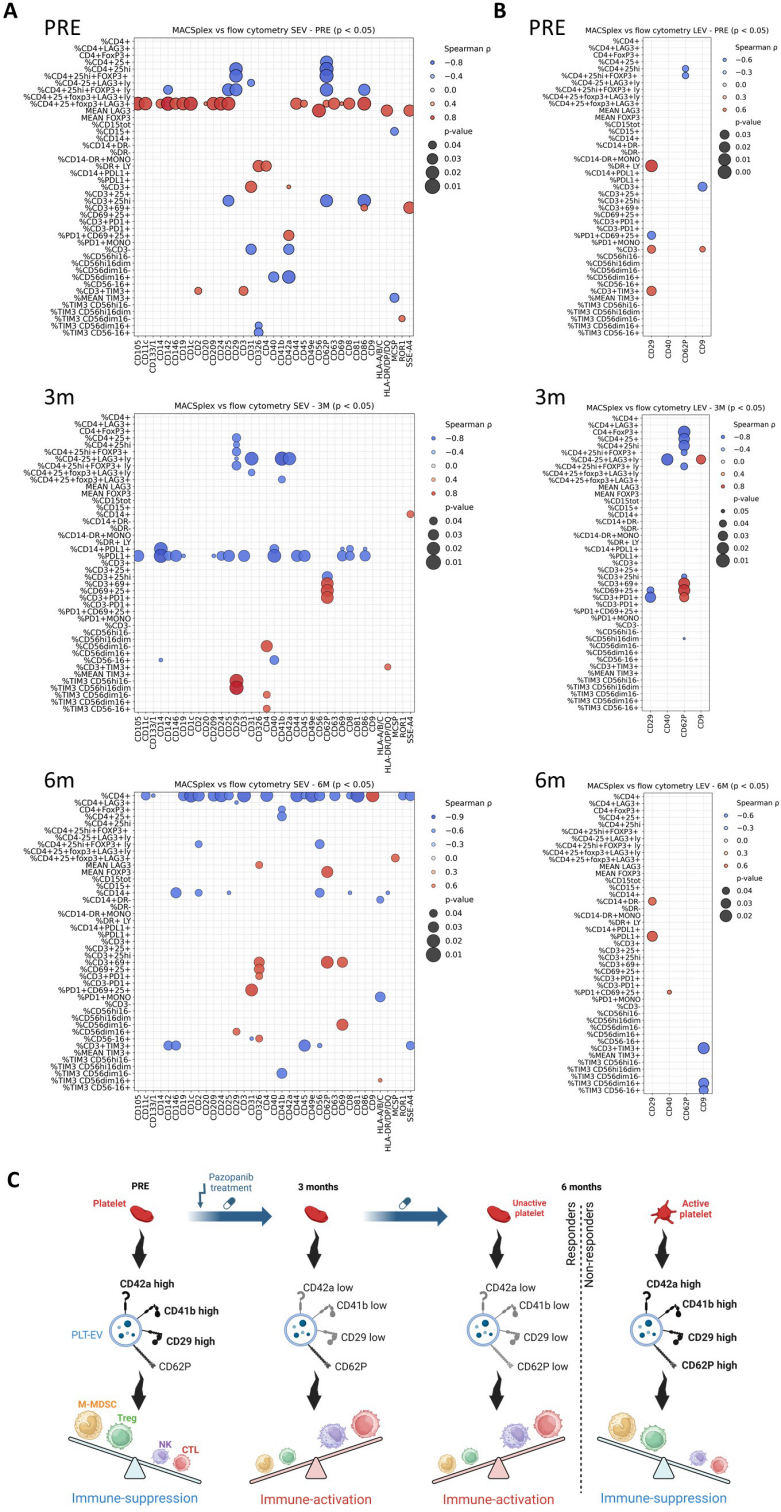
Association between changes in circulating immune cells and EVs markers under Pazopanib treatment. **(A, B)** Bubble plots representing positive (red) and negative (blue) Spearman correlations of SEVs **(A)** and LEVs **(B)** markers with flow cytometry before (PRE) and after three (3M) and six (6M) months of treatment. Bubble size is proportional to the p value. **(C)** Schematic model illustrating the temporal relationship between platelet-derived extracellular vesicle (PLT-EV) phenotypes and immune modulation during Pazopanib treatment. Our results suggest that early treatment (three months) limits the co-expression of key platelet markers on CD62P+ EVs, potentially reducing their immunosuppressive impact on PBMCs. By six months, platelets activation, together with increased levels of CD29, CD41b, CD42a and CD62P on EVs, may restore immunosuppressive interactions and contribute to the onset of Pazopanib resistance.

In order to evaluate the role of PLT-EVs in the PLT-PBMC interaction, we correlated the results of the flow cytometry analysis of the PBMCs obtained from the treated patients with their matched MACSplex EV profiles. Spearman correlation analysis revealed multiple time-dependent associations between PLT markers on EVs and immune cell populations (**Figures 4A, B**). At early timepoints, EVs carrying PLT markers negatively correlated with CD4^+^FoxP3^+^ regulatory T cells: in SEVs CD62P pre-treatment, CD31 at three months and CD41b at six months; in LEV CD62P at pre- and three-month timepoints. Only CD62P on SEVs exhibited positive correlations with the CD4^+^CD25^+^FoxP3^+^LAG3^+^ subset pre-treatment and with FoxP3 MFI at six months (**Figure 4A**). Notably, FoxP3 MFI was increased in non-responders compared to responders at six months (**Supplementary Figure S10**). SEV-associated PLT markers also correlated positively with activated PD1^+^CD69^+^CD25^+^ T cells pre-treatment (CD42a) and at six months (CD31). CD62P expression on both SEVs and LEVs was associated with CD69^+^CD25^+^, CD3^+^PD1^+^ T cells at three months, while its expression on SEVs also correlated with CD3^+^CD69^+^ populations at three and six months. Conversely, PLT markers in SEVs negatively correlated with NK cell subsets, including CD56^dim^CD16^+^ cells pre-treatment (CD42a) and TIM3^+^CD56^dim^CD16^-^ (CD41b) and CD56^-^CD16^+^ (CD31) populations at six months. Also, CD62P in LEVs was inversely correlated with CD56^high^CD16^dim^ at three months. Altogether, these results are consistent with the hypothesis that PLT-EVs may deliver inhibitory signals that selectively affect NK cytotoxicity at these timepoints.

Interestingly, CD29 in LEVs positively correlated with CD14^+^HLA-DR^-^ M-MDSCs and with PD-L1^+^ monocytes only at six months, while showing negative associations with activated T cells pre-treatment and at three months. SEV-associated CD29 negatively correlated with CD4^+^FoxP3^+^ Tregs at pre- and three-month timepoints and with CD4^+^LAG3^+^ T cells at six months, while positively correlating with NK subsets pre-treatment and CD56^dim^CD16^+^ NK cells at six months. These results support the assumption that CD29 upregulation may enhance PLT-EV docking and uptake (33), allowing the functional reprogramming of target immune cells.

Taken together, our findings indicate that the increasing adhesion and uptake competence (CD29/β1 integrin), PLT activation markers (CD62P) and angiogenic/immunomodulatory cargo (CD105, CD209, CD40) of PLT-EVs at six months may facilitate selective docking and functional delivery to monocytes and CD4^+^ T cells. Notably, PLT-EVs can directly induce FoxP3^+^ Tregs (65) and inhibit their conversion towards the proinflammatory Th17 phenotype (66). Moreover, CD62P has been found to foster the interaction of activated PLT with circulating monocytes exacerbating chronic inflammation (36, 37, 67). The positive correlations observed at six months between CD62P^+^ SEVs and FoxP3 MFI and between CD29^+^ LEVs and M-MDSCs/PD-L1^+^ cells are consistent with a mechanistic model in which the emerging immunosuppression is influenced by PLT-EVs uptake, thanks to enhanced selective targeting (**Figure 4C**).

## 4 Conclusions

Our longitudinal analysis of plasma EVs in mRCC patients undergoing antiangiogenic therapy reveals a biphasic remodeling of the PLT-EV compartment, closely associated to immune reprogramming. At three months, transient reductions in PLT markers (CD41b, CD42a) coincided with systemic immune activation, reduced monocytic MDSCs and enhanced NK and T cell cytotoxicity, suggesting a common effect of PLT and immune cells reprogramming. By six months, however, the onset of resistance is accompanied by PLT activation and the rebound of circulating PLT-EV, which show a complex phenotype characterized by an increased adhesion capacity (CD29/β1 integrin, CD62P/P-selectin) and immunomodulatory or angiogenic cargo (CD105, CD209, CD40).

Transcriptomic analysis of PBMCs further supported these dynamics: in responders, GSEA revealed a downregulation of PLT activation, aggregation and PDGF signaling pathways, together with sustained activation of T cell differentiation and antigen receptor signaling, compared to non-responders. These findings suggest that Pazopanib efficacy is associated with reduced PLT activation signatures within PBMCs, reflecting functional PLT-immune cross-talk that influences treatment response.

Correlation analyses support a model in which reprogrammed PLT-EVs selectively dock to and modulate immune cells: promoting PD-L1 upregulation on monocytes, favoring M-MDSC and Treg expansion and suppressing NK cytotoxic subsets.

Our results identify PLT-EVs as possible dynamic mediators of the immunological remodeling under Pazopanib therapy, which takes part in the transition from early immune activation to later immune suppression. By integrating adhesion molecules, activation markers and immunomodulatory ligands, PLT-EVs emerge as potential mediators of angiogenic and suppressive signaling contributing to resistance. This highlights the possibility that PLT-EVs may represent not only a useful source of biomarkers associated with Pazopanib response but also potential targets with cross-cancer translational potential (68). Disruption of PLT activation and PLT-immune cross-talk by EV-like carriers for improved stability and on-demand activation or therapeutic-EV applications (69) may enhance the durability of TKI-induced immune activation. However, studies in larger cohorts as well as *in vitro* mechanistic assays, including co-culture and neutralization assays, are required to confirm these hypotheses and determine whether therapeutic modulation of PLT-(EVs) may contribute enhancing the durability of TKI-induced immune activation in RCC patients.

## Data Availability

The raw data supporting the conclusions of this article will be made available by the authors, without undue reservation.
