# Supplementary material for: Pazopanib-associated remodeling of platelet-immune cell crosstalk and immune suppressive platelet-derived extracellular vesicles in metastatic RCC
Source: Front Immunol. 2026 Jan 7;16:1696460. doi: 10.3389/fimmu.2025.1696460 (PMC12819826; doi:10.3389/fimmu.2025.1696460)
Supplement: Supplementary file 1 [file Presentation1.pdf]

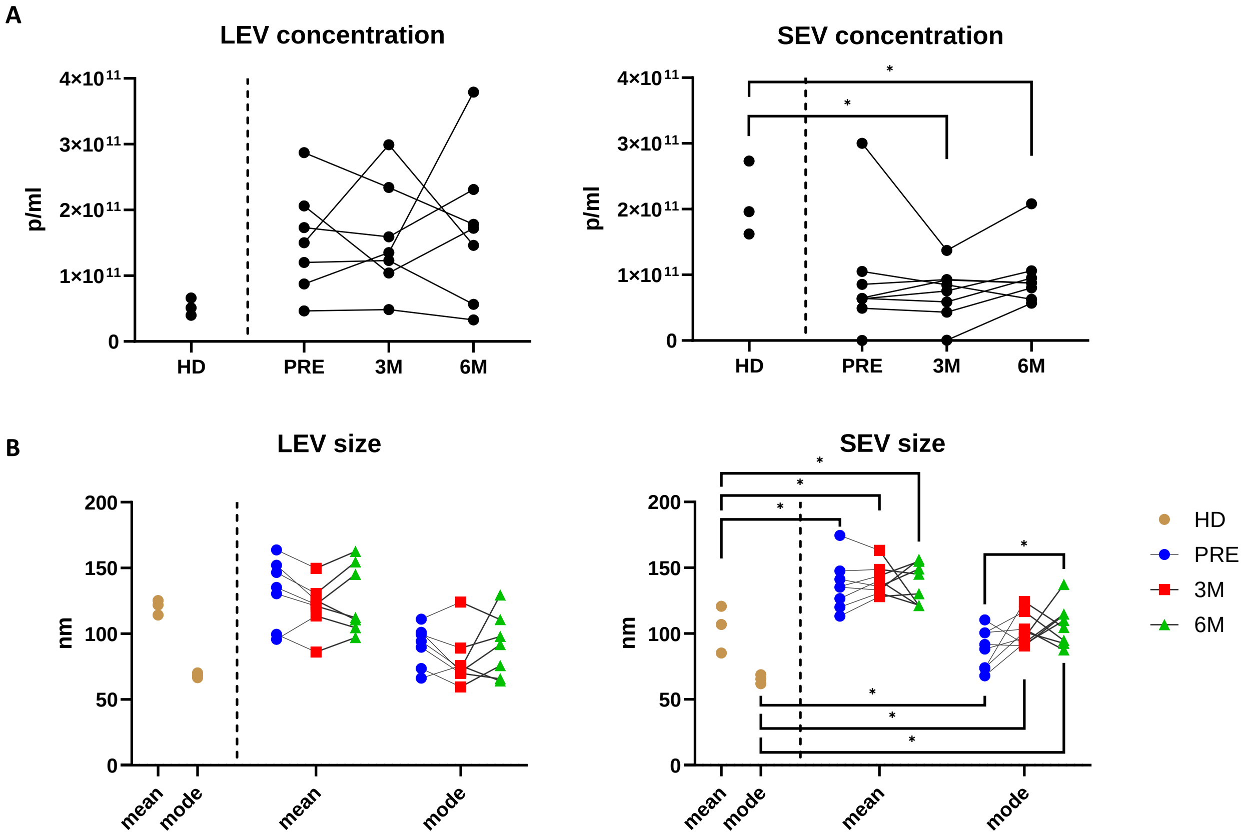

Figure S1. Nanoparticle tracking analysis of EVs from patients at baseline and on Pazopanib therapy at 3 and 6 months (n=8) and healthy donors (n=3). (A) Particle concentration of the obtained SEV and LEV fractions from plasma of patients before and after treatment and from healthy donors. (B) Mean and mode of particle dimensions in the two EVs fractions LEV and SEV deriving from patients and healthy donors (HD). Statistical significance was achieved with Mann-Whitney test (HD vs patients) and Wilcoxon paired t-test (longitudinal analysis patients), * $p < 0.05$.

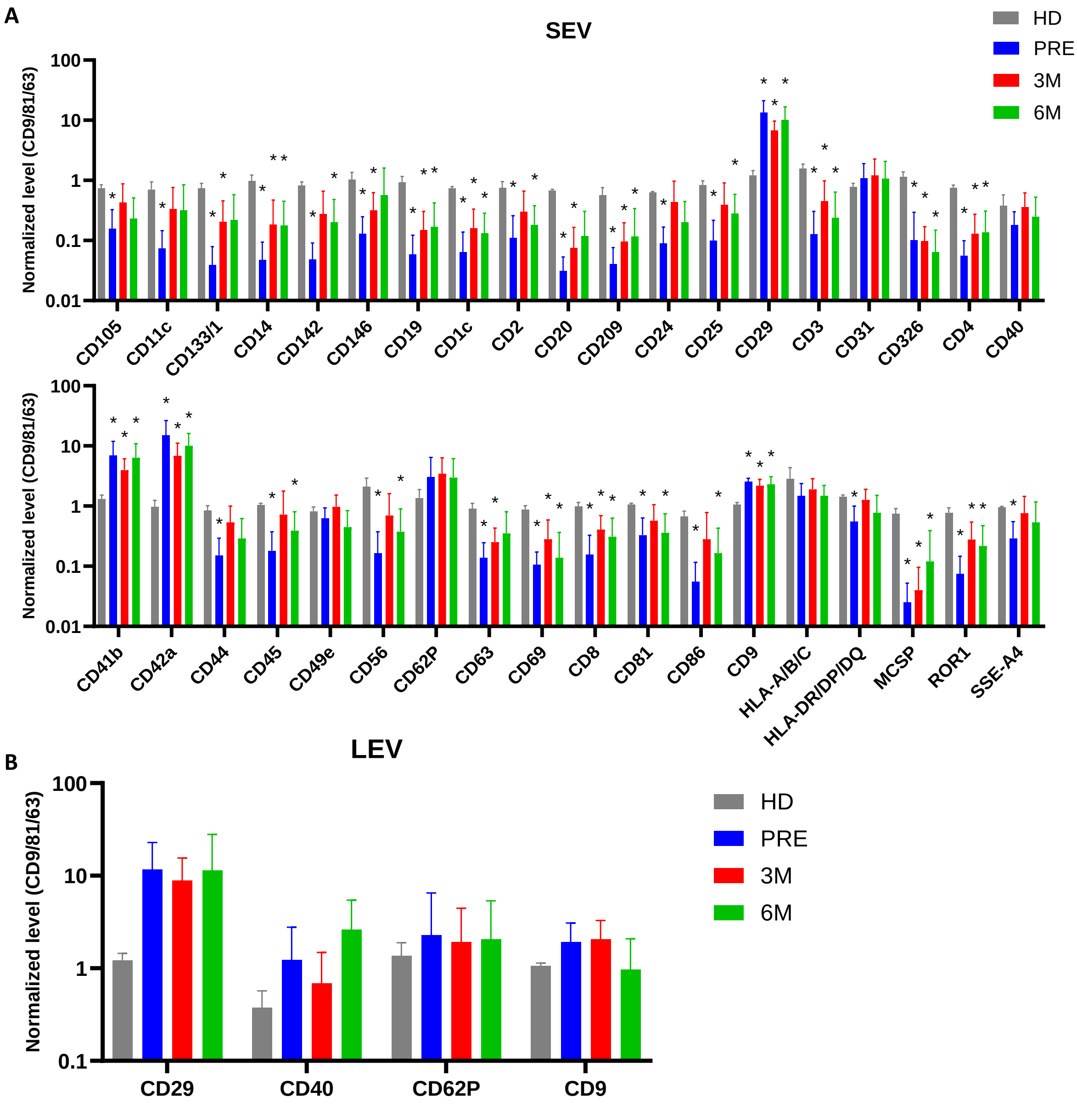

Figure S2. (A, B) Changes in EV markers on patient-derived SEVs and LEVs after Pazopanib treatment compared to healthy donors (n=3). The results are expressed as $\log_{10}$ of values normalized to the EV-related markers CD9, CD81, and CD63. Significance compared to healthy donors is shown (Mann-Whitney test): * $p < 0.05$.

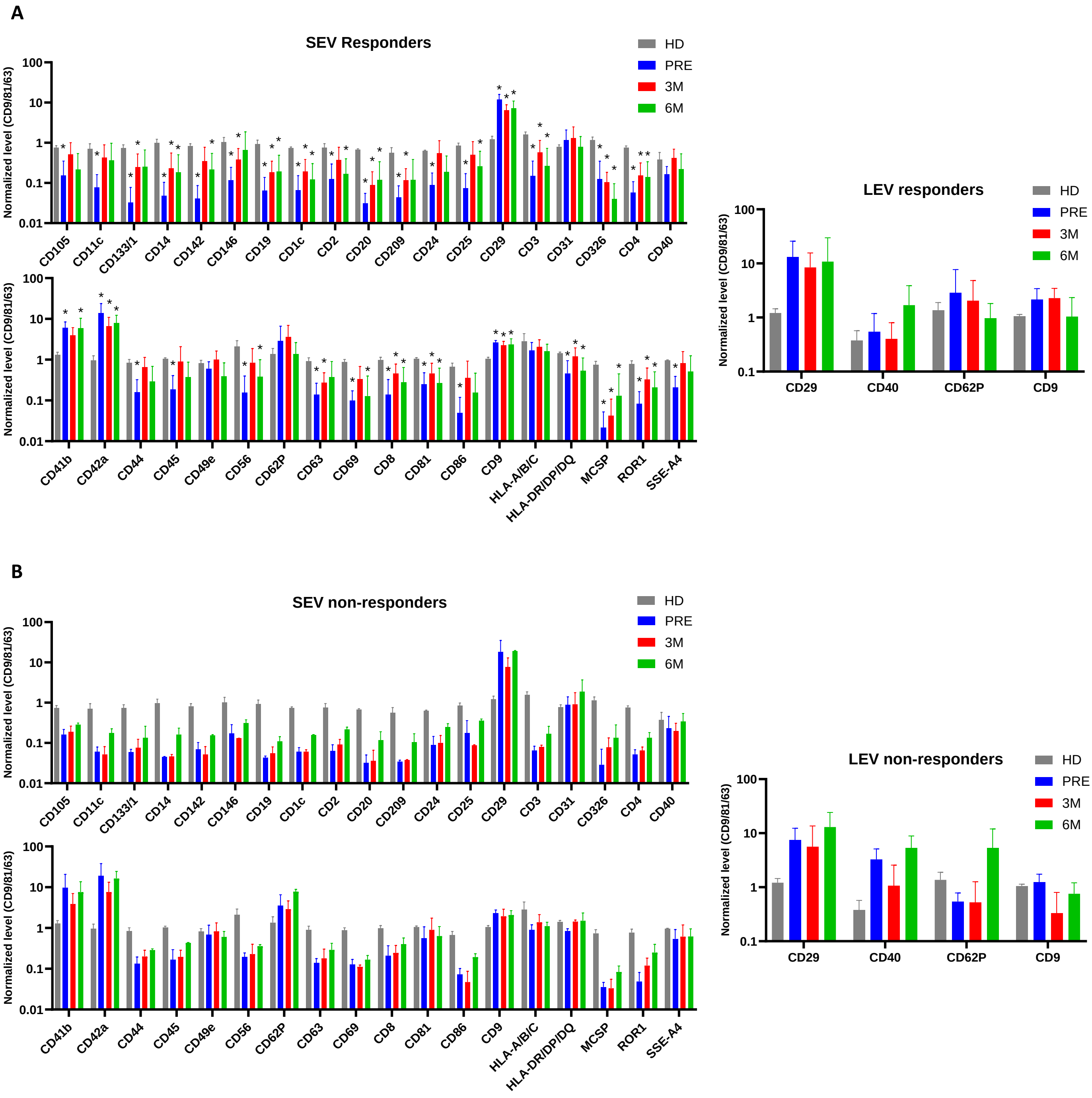

Figure S3. (A, B) MACSplex results of SEVs and LEVs markers from responders (n=6) (A) or non-responders (n=2) (B) after Pazopanib treatment compared to healthy donors (n=3). The results are expressed as $\log_{10}$ of values normalized to the EV-related markers CD9, CD81, and CD63. Significance compared to healthy donors is shown (Mann-Whitney test): * $p < 0.05$.

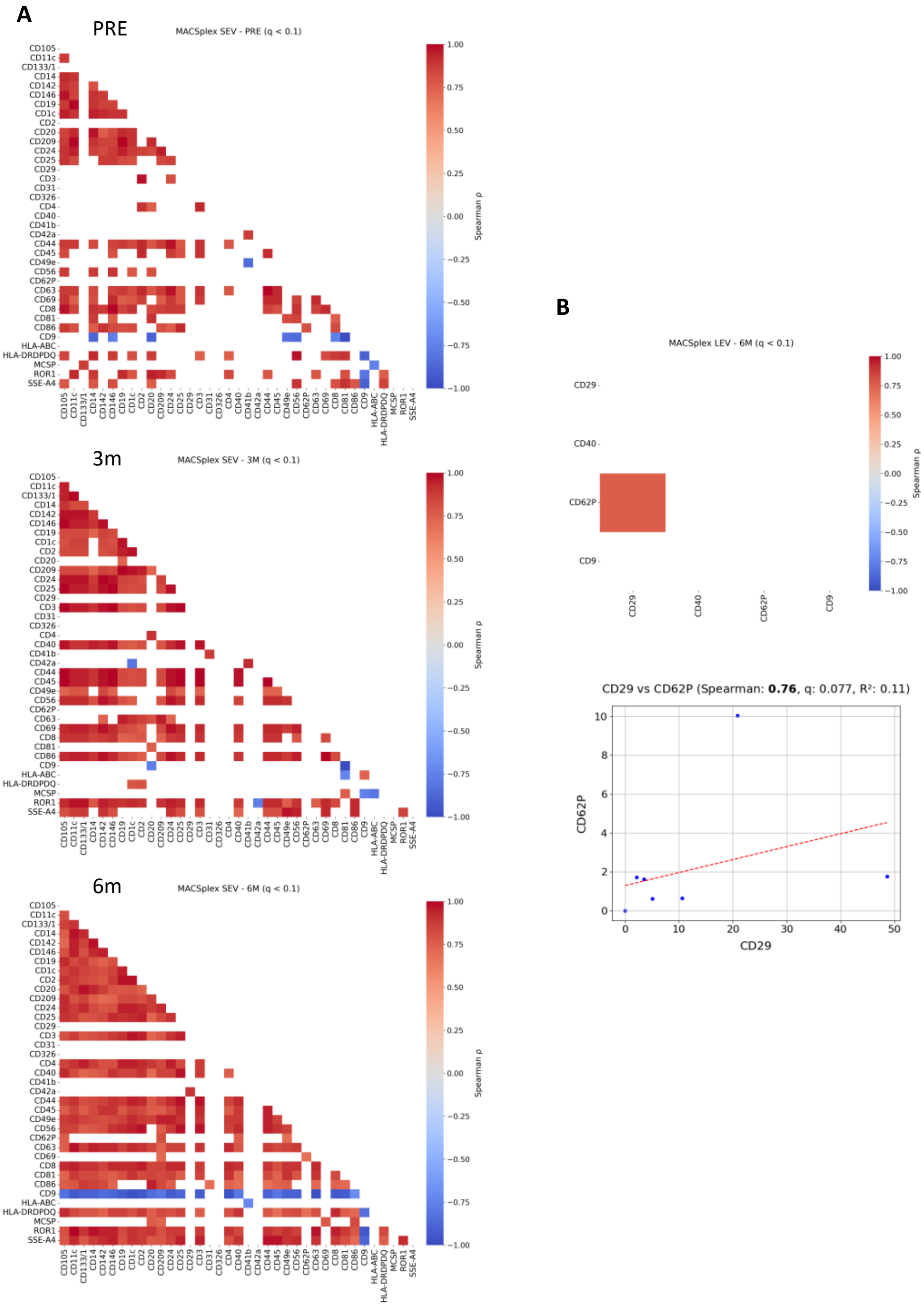

Figure S4. Correlation analysis of EV surface markers (A) Spearman correlation matrix of the negative (blue) and positive (red) associations among SEVs markers. Only results with q value < 0.1 are shown. (B) The correlation between CD62P and CD29 in LEVs after six months of treatment is shown in a matrix (upper) and a scatter plot (lower).

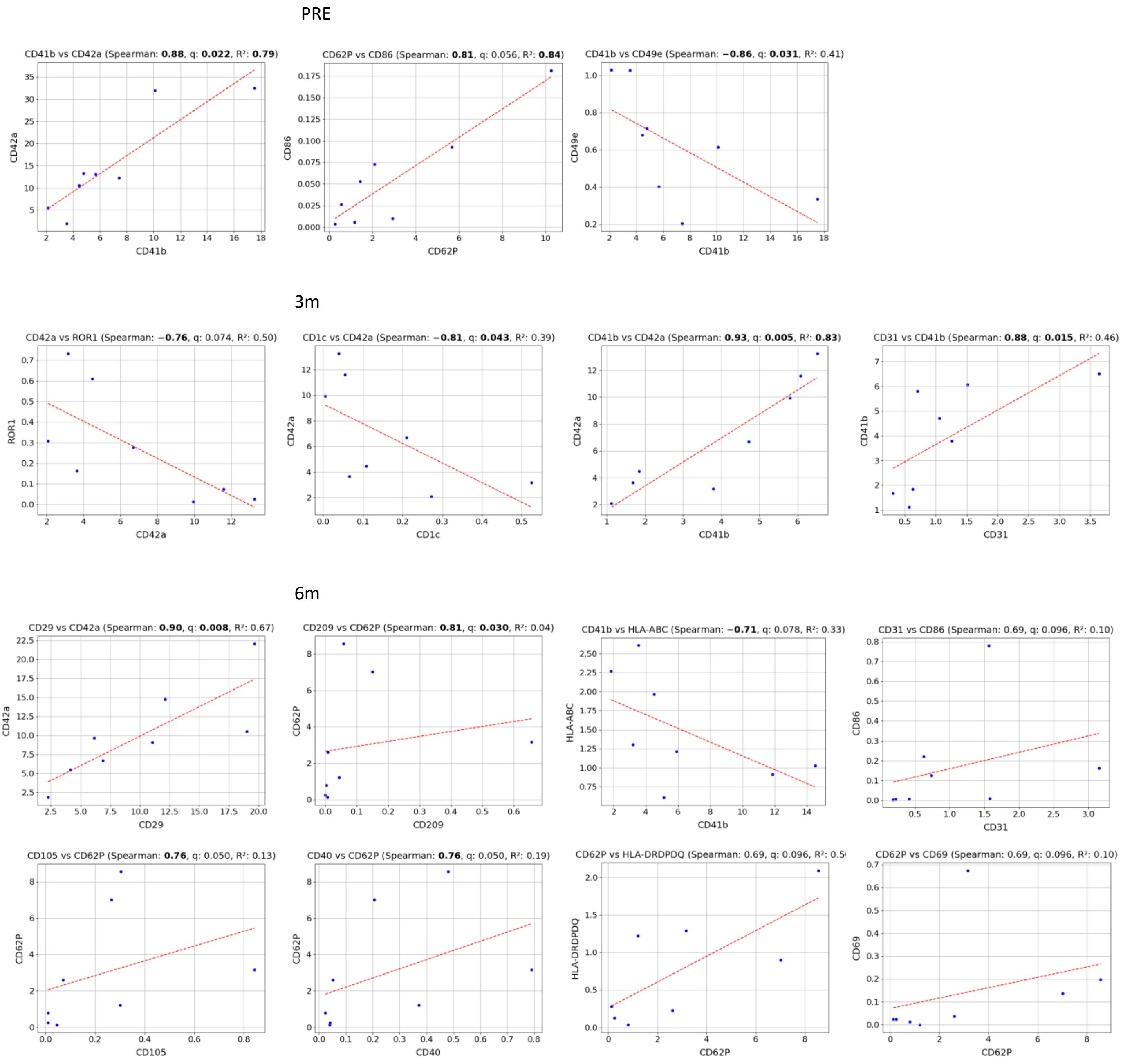

Figure S5. Scatter plots of the significant correlated PLT-markers on SEVs (q < 0.1).

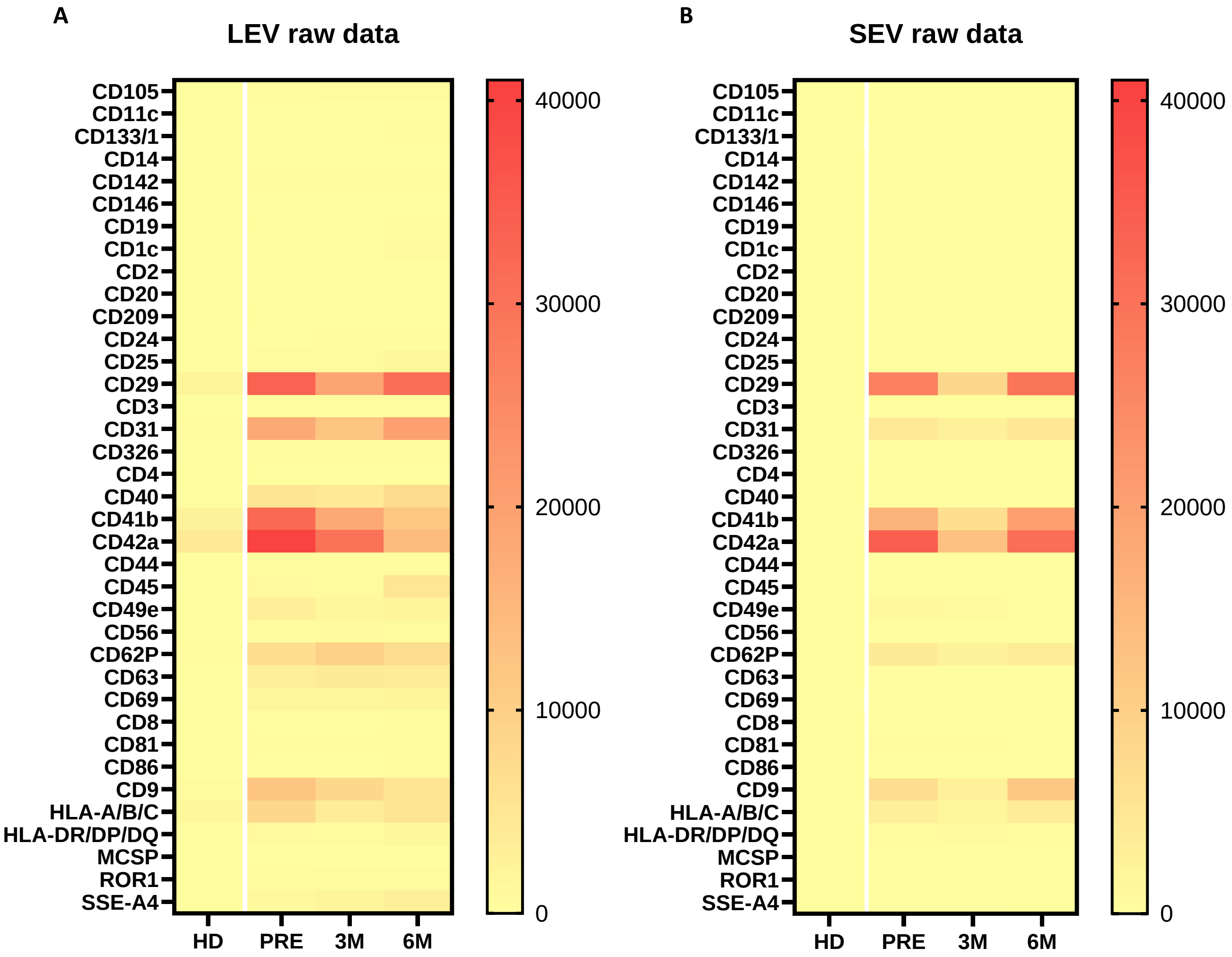

Figure S6. Effects of Pazopanib Treatment on EV surface markers without normalization. (A, B) Heatmap showing the levels of 37 EV markers on LEVs and SEVs of mRCC patients (n=8) before and after three and six months of Pazopanib treatment, compared to healthy donors (n=3). Levels are shown as a gradient from yellow to red.

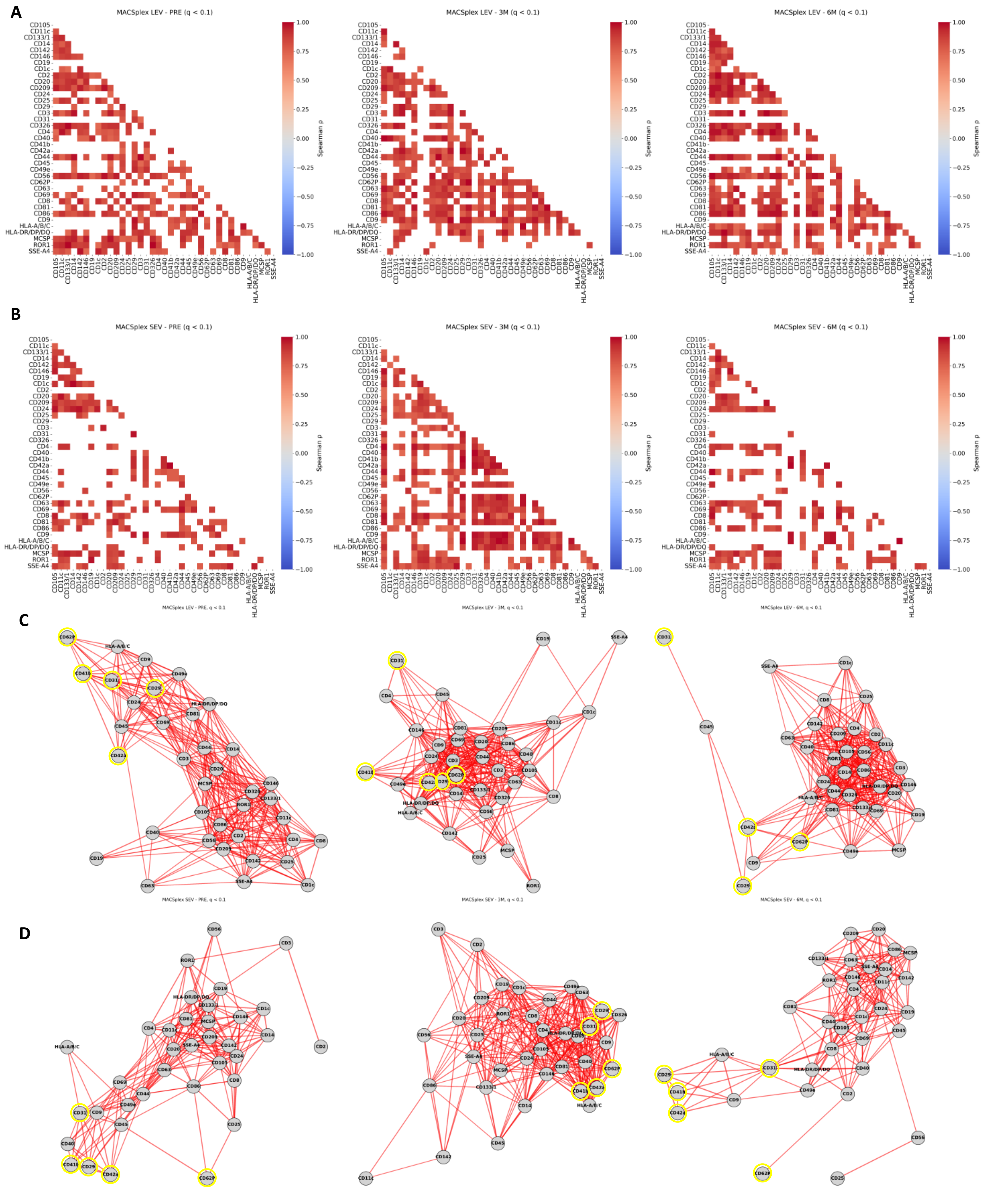

Figure S7. Correlation analysis of EV surface markers without normalization (A) Spearman correlation matrix of the negative (blue) and positive (red) associations among LEVs markers. Only results with q value < 0.1 are shown. (B) Spearman correlation matrix of the negative (blue) and positive (red) associations among SEVs markers. Only results with q value < 0.1 are shown. (C) Spearman correlation network diagram showing significant correlations between LEV markers. Negative and positive correlations between nodes are shown as red and blue lines, respectively. PLT related markers are indicated by yellow circles. (D) Spearman correlation network diagram showing significant correlations between SEV markers. Negative and positive correlations between nodes are shown as red and blue lines, respectively. PLT-related markers are indicated by yellow circles.

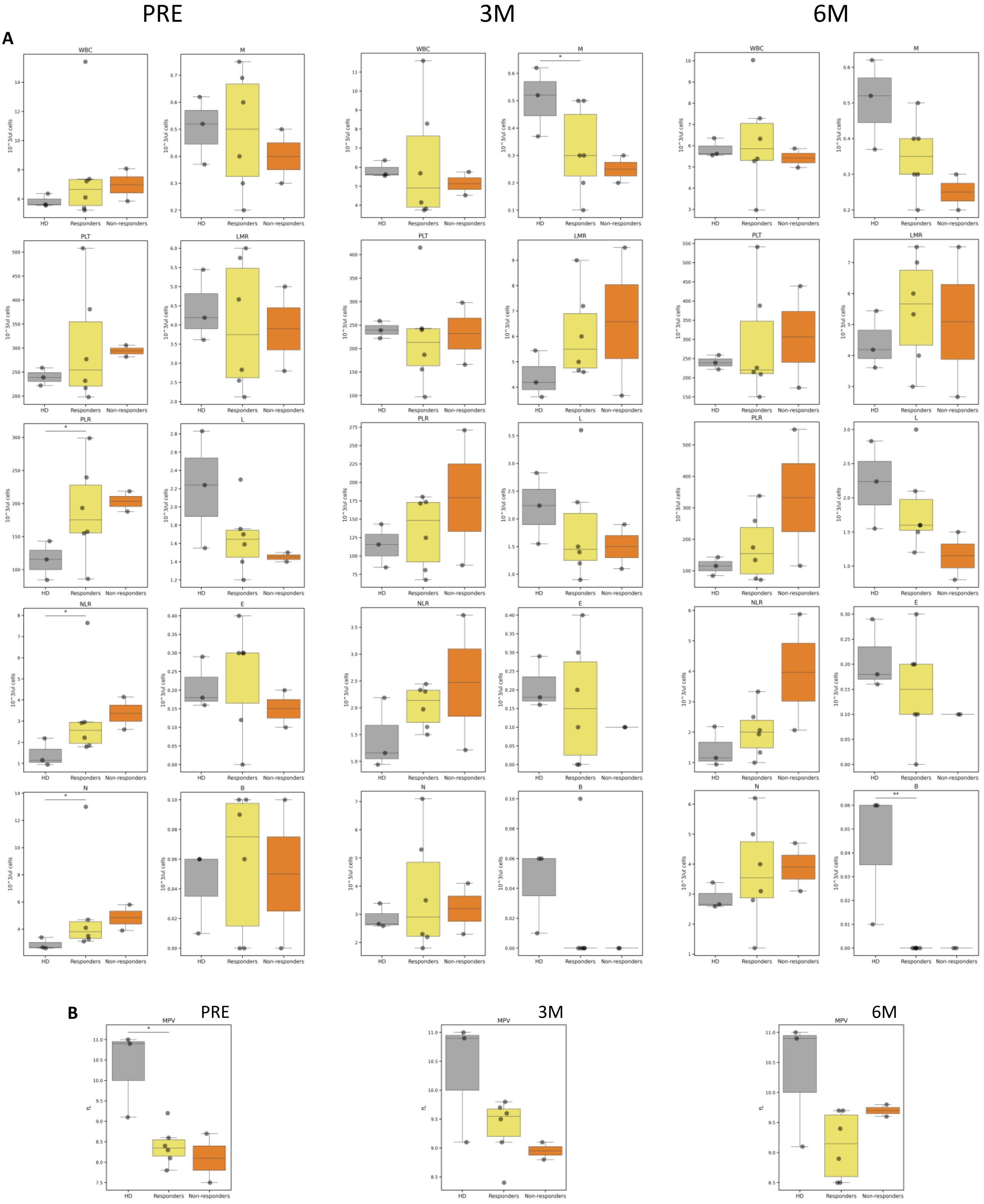

Figure S8. Blood count (A) and MPV (B) differences between responders, non-responders and healthy donors after Pazopanib treatment. Mann-Whitney test: * p < 0.1.

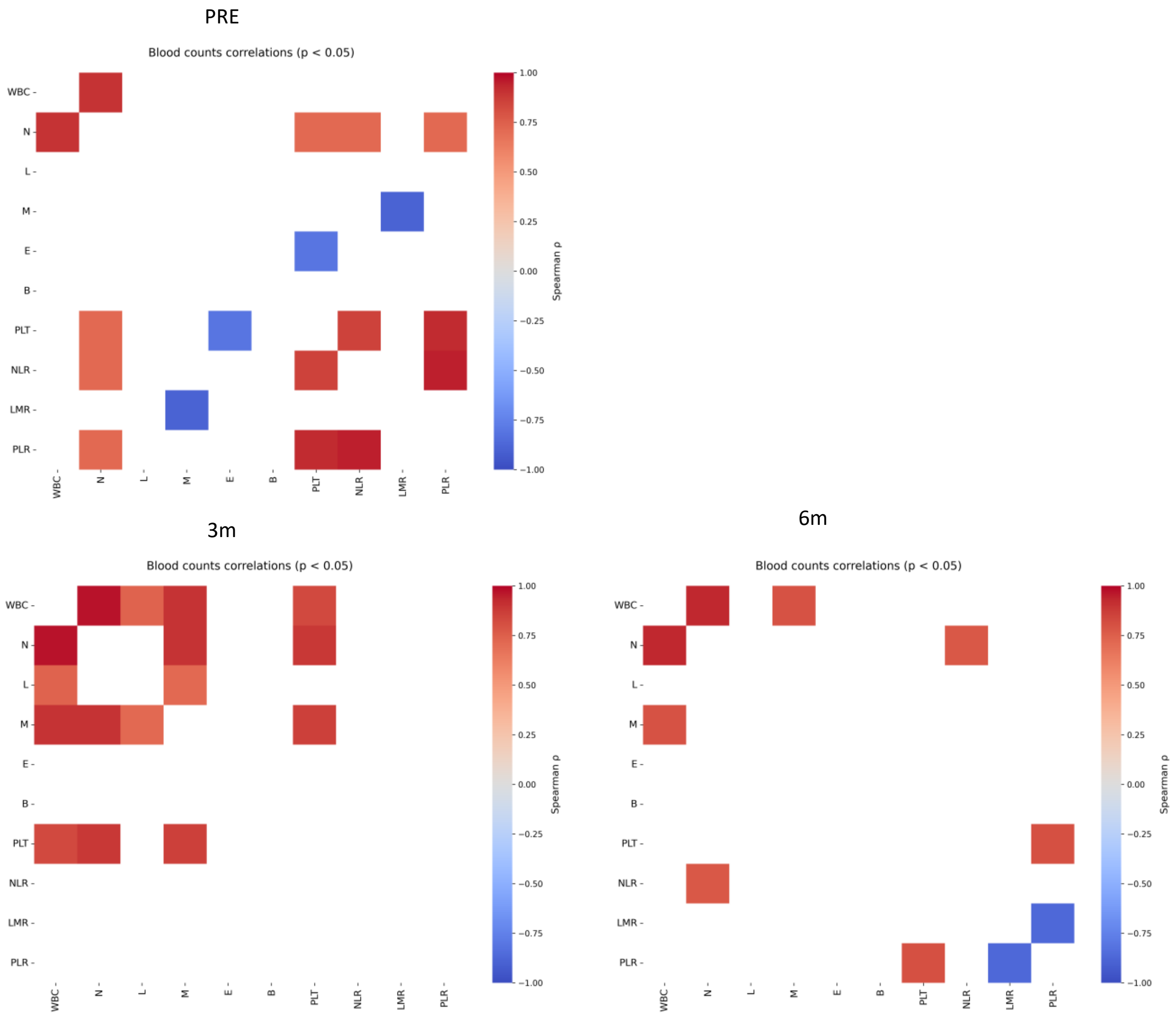

Figure S9. Heatmaps of the significant correlations among blood cell counts. Only results with p < 0.05 are shown.

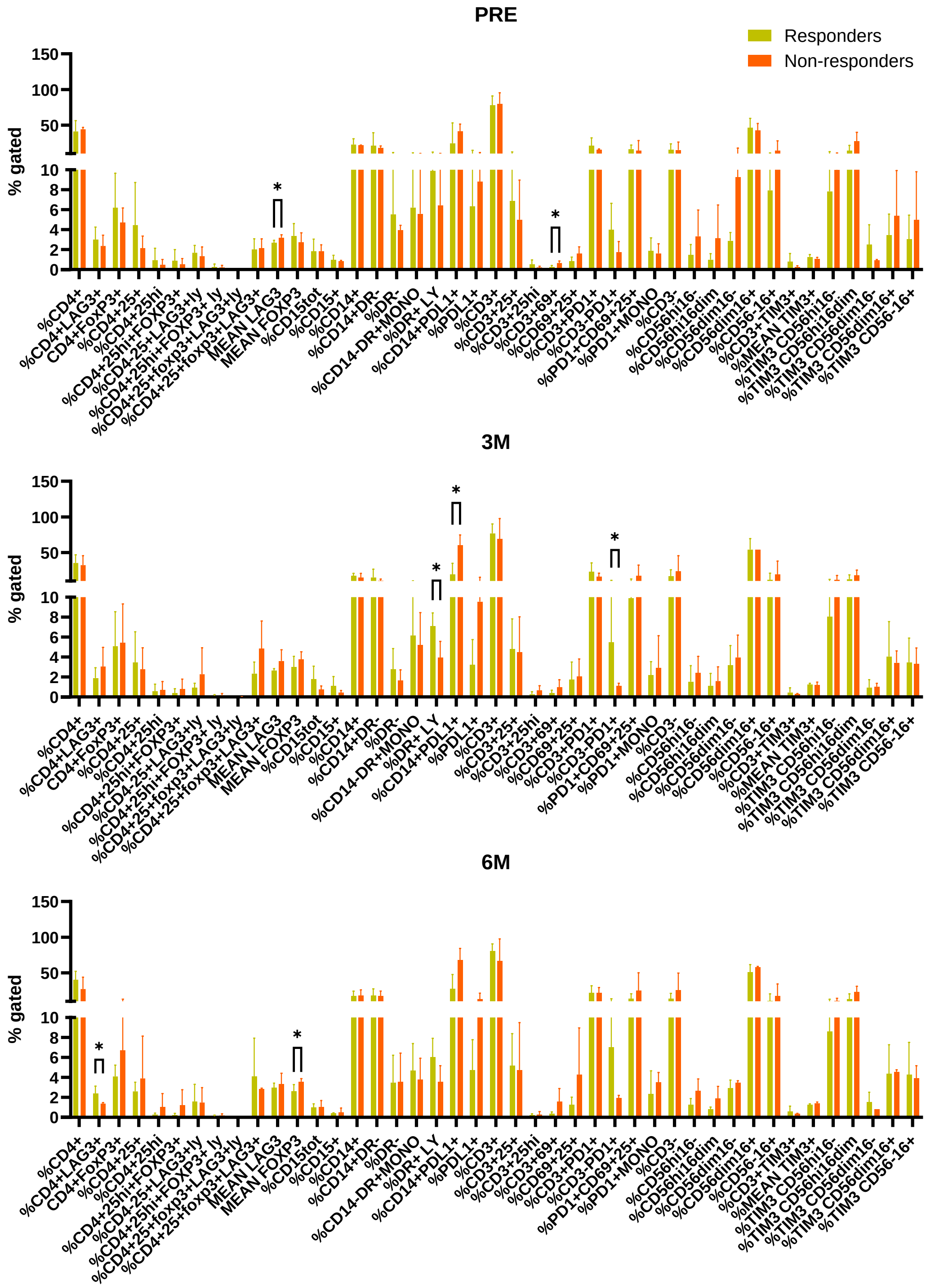

Figure S10. Flow cytometry results of responder (n=6) and non-responder (n=2) groups under Pazopanib treatment. Mann-Whitney test: * $p < 0.1$
